# The effect of dexmedetomidine on perioperative events of orthognathic surgery: a systematic review of randomized controlled trials

**DOI:** 10.4317/medoral.27260

**Published:** 2025-05-27

**Authors:** Caio Melo Mesquita, Aurélio Carlos Diniz, Silvio Pedro da Silva Sakamoto, Walbert A Vieira, Rui Barbosa de Brito-Junior, Marcelo Dias Moreira de Assis Costa, Luiz Renato Paranhos

**Affiliations:** 1 ORCID: 0000-0002-6892-3659. Post-Graduation Program, School of Dentistry. Federal University of Uberlândia - UFU / MG, Brazil; 2 ORCID: 0009-0002-2007-8924. School of Dentistry. Federal University of Uberlândia - UFU / MG, Brazil; 3 ORCID: 0000-0003-3736-3473. Post-Graduation Program, School of Dentistry. University of Rio Verde - UniRV / GO, Brazil; 4 ORCID: 0000-0001-8872-2865. Associate Professor, DDS, MSc, PhD. Department of Dentistry, Endodontics Division. University Center of Associated Schools of Education - UNIFAE / SP, Brazil; 5 ORCID: 0000-0002-3484-9438. Associate Professor, DDS, MSc, PhD. Faculty of São Leopoldo Mandic / SP, Brazil; 6 ORCID: 0000-0001-9148-3674. Associate Professor, DDS, MSc, PhD. Department of Dental Clinic, Oral Pathology and Surgery. School of Dentistry. Federal University of Minas Gerais - UFMG / MG, Brazil; 7 ORCID: 0000-0002-7599-0120. Associate Professor, DDS, MSc, PhD. Division of Social and Preventive Dentistry. School of Dentistry. Federal University of Uberlândia - UFU / MG, Brazil

## Abstract

**Background:**

Orthognathic surgery is a complex invasive procedure associated with common postoperative symptoms and patient-related events. Dexmedetomidine is an emerging sedative and hypotensive agent that has demonstrated safety and efficacy in perioperative care of other craniofacial procedures.

**Material and Methods:**

An electronic search was performed in seven primary databases (Cochrane Library, Embase, LILACS, MedLine via PubMed, SciELO, Scopus, and Web of Science) and one additional (EASY) to partially capture the gray literature. The PICO strategy was used to identify randomized clinical trials evaluating the effect of dexmedetomidine on perioperative events in patients undergoing orthognathic surgery compared to placebo or control groups, without restrictions on publication language and year. Two independent reviewers performed data extraction and assessed the risk of bias using the RoB 2.0 tool.

**Results:**

The search identified 401 records, of which six studies met the eligibility criteria, including 282 patients from five countries, and published between 2008 and 2023. Outcomes were categorized into six groups based on available data: 1) Airway and Respiratory Events, 2) Emetic Events, 3) Hemodynamic Events, 4) Length of Hospital Stay, 5) Neurological Events, and 6) Pain Burden. Dexmedetomidine reduced coughing and maintained hemodynamic stability but did not prevent emergence agitation. It was associated with lower intraoperative fentanyl use and reduced rescue analgesia requirements. Postoperatively, dexmedetomidine effectively controlled pain, nausea, and vomiting, with significantly lower pain scores and reduced analgesic demand. Among the six studies, only one was classified as high risk of bias due to issues in the randomization process, while the others were categorized as low risk of bias. A meta-analysis was planned but could not be conducted due to high heterogeneity among studies.

**Conclusions:**

Dexmedetomidine appears to be a safe and effective option for reducing postoperative symptoms such as pain, nausea, vomiting, and cough in orthognathic surgery, while maintaining hemodynamic stability.

** Key words:**School of Dentistry, Federal University of Uberlândia Campus Umuarama, Av. Pará, nº 1720, Block 2G, room 1 Postal Code 38405-320, Uberlândia, MG, Brazil

## Introduction

Nausea, vomiting, pain, and edema are common postoperative symptoms following orthognathic surgery, significantly impacting patient recovery and quality of life (1-3). Despite advancements in surgical and perioperative care, there is still a lack of standardized protocols to prevent or mitigate these symptoms. Multimodal strategies, such as the Enhanced Recovery After Surgery (ERAS) protocol, have shown promise but remain underexplored in the context of orthognathic surgery (1).

Elements of the ERAS protocol, including systemic and nonsystemic perioperative therapies, have been studied in this surgical field. However, the heterogeneity of studies and variability in reported outcomes—such as postoperative symptoms, complication rates, and length of hospital stay—highlight the need for more robust and consistent evidence to guide its application (3,4).

Dexmedetomidine, a sedative and hypotensive agent, has emerged as a potentially valuable option for perioperative care. It has been proven effective and safe in reducing postoperative emergence delirium in pediatric dental patients (5) and has shown safety in various craniofacial surgeries, including craniotomy (6), endonasal procedures (7), nasal surgeries (8), ophthalmic (9), and middle ear surgeries (10). Moreover, its use in induced hypotensive anesthesia for orthognathic surgery has demonstrated benefits such as reduced intraoperative blood loss and shorter hospital stays (11).

However, the use of dexmedetomidine is not without risks. Prolonged extubation time and an increased likelihood of cardiovascular complications have been reported when it is used as an opioid substitute in opioid-free anesthesia (12). Despite these limitations, its potential benefits in managing postoperative symptoms make it a promising candidate for further investigation.

This study aims to systematically review the literature on the effect of dexmedetomidine on perioperative events of orthognathic surgery, contributing to evidence-based recommendations for improving patient outcomes.

## Material and Methods

- Protocol registration

The protocol was reported according to the Preferred Reporting Items for Systematic Review and Meta-Analysis Protocols (PRISMA-P) (13) and registered in the International Prospective Register of Systematic Reviews (PROSPERO) database under number CRD42024527967 (https://www.crd.york.ac.uk/PROSPERO/). This systematic review was reported following the Preferred Reporting Items for Systematic Reviews and Meta-Analyses (PRISMA) (14) and conducted according to the Joanna Briggs Institute (JBI) Manual.

- Research question and eligibility criteria

The review was designed to answer the following question: "Does the use of dexmedetomidine enhance perioperative events of orthognathic surgery?" following the PICO framework: *P* (population), I (intervention), C (comparison), and O (outcome).

The inclusion criteria were: (1) patients undergoing orthognathic surgery treatment, without restrictions for Angle classification or type of surgical approach; (2) hypotensive anesthesia using dexmedetomidine; (3) placebo and other treatments (e.g. clonidine, saline solution, nitroglycerin, etc.); (4) perioperative events (i.e., airway/respiratory, emetic, and hemodynamic events, length of hospital stays, neurological events, and pain burden); (5) randomized clinical trials; (6) there were no restrictions on publication language or year.

The exclusion criteria were: (1) studies without a control group; (2) studies with overlapping samples (e.g. same authors and samples, but different years and journals of publication), in these cases being considered the most recent study that best describes methodology and results; (3) wrong study or publication type (e.g., books, book chapters, case reports, case series, event annals, editorials, letters to the editor, literature reviews, qualitative studies and animal studies).

- Sources of information, search, and selection of studies

The electronic searches were performed on December 2023 in Cochrane Library, Embase, LILACS (Latin American and Caribbean Health Science Literature), MedLine (via PubMed), and SciELO; and the Scopus and Web of Science citation databases. The EASY database partially captured the "gray literature." These steps were performed to minimize the selection bias. The MedLine search was constantly updated with electronic alerts until January 2025. The search descriptors were selected according to the MeSH (Medical Subject Headings), DeCS (Health Sciences Descriptors), and Emtree (Embase Subject Headings) resources. The Boolean operators "AND" and "OR" promoted several combinations among the descriptors, respecting the syntax rules of each database. Table 1 shows more details of search strategies and databases.

The obtained results were exported to the EndNote Web™ software (Clarivate™ Analytics, Philadelphia, USA), in which duplicates were removed automatically, and the remaining ones were removed manually. The other results were exported to Rayyan QCRI (Qatar Computing Research Institute, Doha, Qatar) (15) for the study selection phase. The manual analysis of the gray literature occurred simultaneously and fully using Microsoft Word™ 2010 (Microsoft™ Ltd., Washington, USA).

Before selecting the studies, two reviewers performed a calibration exercise in which they discussed the eligibility criteria and applied them to a sample of 20% of the retrieved studies to determine inter-examiner agreement. The selection started after reaching an adequate level of agreement (Kappa ≥ 0.81) and occurred in two phases.

In the first phase, two eligibility reviewers (SPSS and ACD) methodically analyzed the titles and abstracts of the studies independently. A third examiner (CMM) investigated and solved disagreements between the reviewers. Titles unrelated to the topic were eliminated in this phase as well as abstracts, respecting the eligibility criteria. In the second phase, the full texts of the preliminarily eligible studies were obtained and evaluated. If the full texts were not found, a bibliographic request was made to the library database (COMUT), and the Correspondence was contacted twice, with a 15-day interval, to obtain the requested texts.

- Data collection

A calibration exercise was performed before data extraction to ensure consistency between the reviewers, in which the data from one eligible study was extracted jointly. After the calibration, two reviewers (SPSS and ACD) extracted the data from the eligible studies, independently and blinded. A third reviewer (CMM) analyzed the conflicts in cases of disagreement about data extraction.

The following data were extracted from the articles: study characteristics (author, year, title, journal of publication, impact factor using Journal Citation Reports 2023, study country, study design, type of randomization, ethical criteria, funding, and conflict of interest), sample characteristics (sample size, sex, age, ethnicity, eligibility criteria, study groups, and Angle class of participants), data collection and processing (perioperative medication protocols, anesthesia protocols, surgical technique, perioperative events assessed, assessment methods, and statistical tests), and main results (objectives, perioperative events, and main outcomes). In case of incomplete or insufficient data, the Correspondences were contacted via e-mail up to three times at weekly intervals.

- Risk of bias assessment

Two reviewers (SPSS and ACD) independently assessed the risk of bias in the selected studies using the Cochrane Collaboration Risk of Bias tool (version 2.0) (RoB2) for RCTs (16). This instrument consists of five domains: bias from the randomization process, bias due to deviations from intended interventions, bias from missing outcome data, bias in outcome measurement, and bias in the selection of reported results.

The evaluation of each domain followed the algorithms proposed by the RoB2 manual (16). Any disagreements between the reviewers were resolved by discussing and consulting with a third reviewer (CMM).

- Summary measures and synthesis of results

The data collected from the selected studies were organized in spreadsheets on Microsoft Excel™ 2019 (Microsoft™ Ltd., Washington, USA) and described narratively (qualitative synthesis). The quantitative results of the use of dexmedetomidine for the reduction of postoperative symptoms after orthognathic surgery were described. A meta-analysis was planned but not performed due to the high heterogeneity of the studies.

- Certainty of evidence (GRADE approach)

Two reviewers (CMM and WAV) independently ranked the overall strength of evidence using the Grading of Recommendations, Assessment, Development, and Evaluation (GRADE) tool (17). To assess the criteria in systematic reviews without meta-analyses, the authors followed the adaptations by Murad *et al*. (18).

## Results

- Study selection

**Figure 1:**
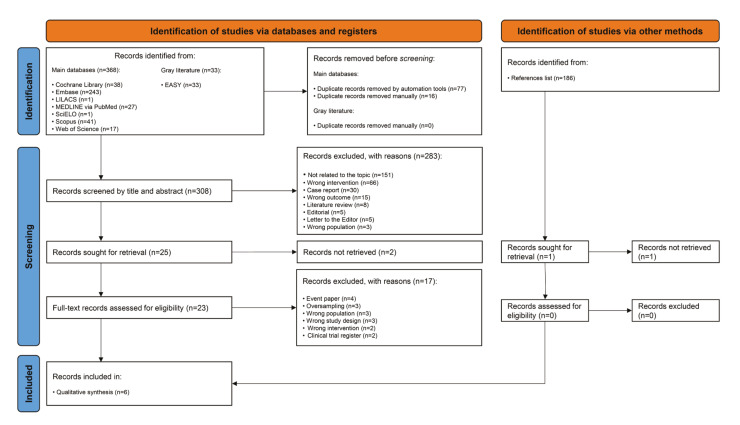
Flow diagram describing the studies selection.

The electronic search identified 401 results distributed into eight electronic databases and the gray literature. After removing 93 duplicates, 308 records were screened by titles and abstracts, resulting in the exclusion of 283 records. A full-text assessment of 25 studies excluded 19 for not meeting eligibility criteria, leaving six studies (19-24) included in the qualitative synthesis. Additionally, one record identified from reference lists could not be retrieved. Fig. 1 provides a detailed overview of the study selection process.

- Study characteristics

The included studies were published between 2008 to 2023 and conducted in five countries: five in Asia (20-24), and one in South America (19). A total of 282 patients participated across all eligible studies. The age range of participants varied from 17 years (19) to 45 years (20,21), with female patients representing the majority of the sample in nearly all studies (19-21,23,24), except for one (22).

Only two studies reported patients’ Angle Class (21,24), and one of them reported craniofacial malformations in the sample (21). Surgical technique was specified on four studies (20,21,23,24), with many variations, such as bimaxillary (20,24), Le Fort I, BSSO, Le Fort I + BSSO (21,23), Le Fort I 2-piece osteotomy + BSSO, Le Fort I osteotomy + BSSO + genioplasty, Le Fort I 3-piece osteotomy + BSSO + genioplasty, Le Fort I 3-piece osteotomy + BSSO + lower anterior subapical osteotomy (23). There were no reports of major difference in surgical procedures between the groups of patients.

All studies utilized various medication protocols for pre- and post-operative management. Pre-anesthetic medications included midazolam (19,23,24) and glycopyrrolate (20,23) combined with rocuronium (20) or atracurium (24). Analgesia protocols varied, using combinations of ketoprofen and tramadol (19), ketorolac and fentanyl (20,23), acetaminophen and diclofenac (24), and meperidine (21). Antiemetics included ondansetron (19,20), dexamethasone (19,24), and metoclopramide (24). Antimicrobial use was specified in two studies with metronidazole and cefazolin (19,24). Local anesthesia with lidocaine and epinephrine was reported in only one study (19). Blood pressure control was managed with esmolol (19,20) and nicardipine (20), while ephedrine was used in one study (23).

As for the hypotensive anesthesia protocol. Induction with dexmedetomidine in the intervention group of four studies (19,21,23,24) was achieved by combining it with propofol and other medications, such as sufentanyl, and pancuronium (19); fentanyl (21,23); midazolam (21); and vecuronium (23). One study induction involved propofol, desflurane, and remifentanil in both groups (20). Other study induction was achieved using only propofol and sevoflurane for all groups, with vecuronium for nasotracheal intubation (22). Maintenance protocols varied: propofol (19,23,24), isoflurane, pancuronium (19), desflurane (20,23), remifentanil, phenylephrine (20), fentanyl (21,23), cis-atracurium, nitrous oxide, sevoflurane (21,22), and vecuronium (23). With all the studies making use of dexmedetomidine for intervention group. Recovery protocols included the suspension of induction drugs across all studies. Prostigmine was reported in one study (19), while remifentanil and glycopyrrolate were used in another (20). Atropine (19,21,24) and neostigmine (20,21,24) were commonly used in three studies each. One study did not provide further details on their recovery protocol, they just stated that the drugs used in preparation and maintenance were suspended (23). Other study did not state any detail on the recovery protocol (22). Regarding the induction and maintenance in control groups the studies often used alternatives like clonidine (19,23), remifentanil (22), nicardipine (22), nitroglycerin (21) or normal saline (20,24) in place of dexmedetomidine.

All studies utilized various protocols for assessing perioperative events. Awakening, extubation and Post-Anesthesia Care Unit (PACU) times were measured per minute in one study (19). Pain was a common symptom evaluated across nearly all studies, using scales such as the Visual Analogue Scale (VAS) (19,24) and the Numeric Rating Scale (NRS) (20,21). Nausea and vomiting were recorded per incidence in two studies (19,24). Emergence agitation was assessed using the Richmond Agitation-Sedation Scale (RASS) in one study (20), which also evaluated cough using a four-point scale, eye opening, and discharge from the operation room per minute. Eye opening time was similarly measured in another study (21), which also included time to follow commands and extubation time per minute. Rescue analgesia was assessed per microgram in one study (23) and included specific measurements for the use of meperidine per milligram in another study (21), and rescue fentanyl in yet another (20). Residual sedation and the use of phenylephrine were specifically noted per incidence in one study (20). Rescue antiemetics were documented in one study (20). One study (24) also included a detailed schedule for pain assessment using the VAS at six postoperative time points (1, 3, 6, 12, 18, and 24 hours).

Table 2 presents more information on the main characteristics of eligible studies.

- Individual results of the studies

Farah *et al*. (2008) (19) found no statistically significant differences between the groups regarding physiological responses or surgery duration when comparing dexmedetomidine and clonidine. Estimated blood loss did not significantly differ between the groups. No significant differences were observed in intra- and postoperative systolic or diastolic blood pressure, body temperature variations, or heart rate. However, all patients in the clonidine group required beta-blockade with esmolol to maintain normal to low heart rates, whereas only a few patients in the dexmedetomidine group required esmolol or atropine due to bradycardia. Both protocols were effective and safe for extended orthognathic surgeries with significant blood loss.

Ham *et al*. (2014) (20) showed that a single dose of dexmedetomidine combined with low-dose remifentanil infusion did not effectively reduce emergence agitation in adults with nasotracheal intubation after orthognathic surgery under desflurane-remifentanil anesthesia, compared to low-dose remifentanil alone. Dexmedetomidine significantly reduced the incidence of coughing without causing respiratory depression and maintained hemodynamic stability during emergence and recovery. Its use was associated with reduced pain in the PACU, delayed eye opening, and a longer discharge time from the operating room, but did not lead to residual sedation in the PACU.

Rummasak & Apipan (2014) (21) demonstrated that dexmedetomidine and nitroglycerin produced distinct heart rate responses, despite being administered through different routes. Dexmedetomidine significantly reduced the intraoperative fentanyl requirement compared to nitroglycerin. Times to eye opening and following commands were longer in the dexmedetomidine group, but extubation time did not differ between the groups. Early postoperative pain at 30 and 60 minutes and the requirement for meperidine were similar between the two groups.

Shin *et al*., (2014) (22) found that remifentanil and dexmedetomidine did not stimulate the sympathetic nervous system during controlled hypotension, whereas remifentanil presented better maintenance of overall autonomic nervous system balance. In contrast, nicardipine was associated with sympathetic nervous system stimulation.

Goswami *et al*. (2022) (23) showed that both dexmedetomidine and clonidine were effective and safe for inducing controlled hypotension and ensuring clear operative field visibility. No statistically significant differences were observed between the groups regarding surgical field quality, duration of surgery, or blood loss. The total drug consumption and the need for rescue analgesia were lower in the dexmedetomidine group. Adverse effects were more frequent in the clonidine group than in the dexmedetomidine group.

Labafchi *et al*. (2023) (24) demonstrated that the administration of dexmedetomidine was effective in controlling postoperative pain, nausea and vomiting in patients undergoing bimaxillary orthognathic surgery compared to placebo. Pain scores were significantly lower in the dexmedetomidine group at all time points. The demand for rescue analgesics was significantly higher in the placebo group. Nausea was reported by nearly half of the patients in the placebo group but was rare in the dexmedetomidine group. Postoperative vomiting was not observed in any participant.

Table 3 shows details of the outcomes of each eligible study. Outcomes were categorized into six groups based on available data: (1) Airway and Respiratory Events, (2) Emetic Events, (3) Hemodynamic Events, (4) Length of Hospital Stay, (5) Neurological Events, and (6) Pain Burden.

- Risk of individual bias in the studies

**Figure 2:**
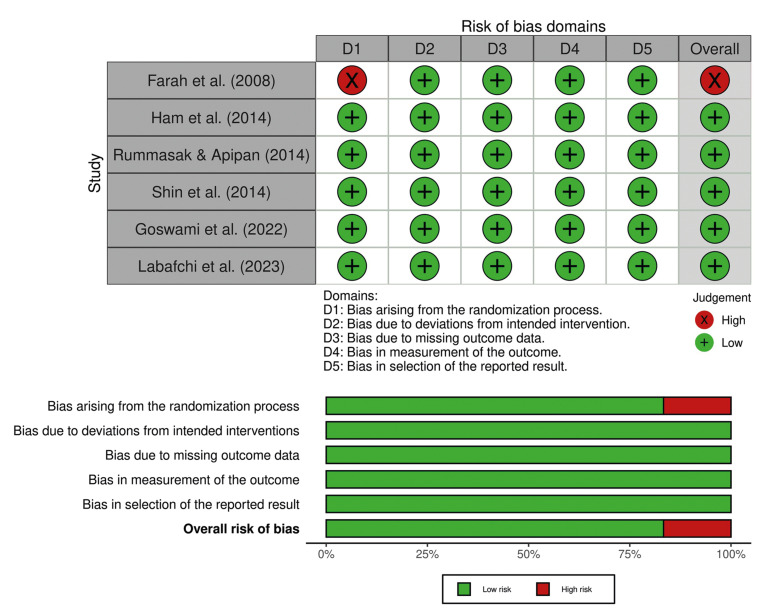
Individual risk of bias assessment.

Among the six studies, only one (19) was classified as a "high risk of bias”, specifically in the domain of bias arising from the randomization process (D1). This was due to insufficient information on the randomization method and allocation concealment. The remaining five studies were classified as “low risk of bias” across all domains. Fig. 2 shows the individual assessment of each included article.

- Certainty of evidence

The certainty of evidence varied from very low to low. The main cause for downgrading the certainty was imprecision (Table 4).

## Discussion

Consistent with previous reviews on other craniofacial procedure (6-10), this systematic review highlights the promising potential of dexmedetomidine as a hypotensive agent in orthognathic surgery. However, the limited number of studies and the heterogeneity among the available randomized clinical trials (19-24) emphasize the need for further high-quality research to establish more definitive conclusions.

The findings of this review also align with the growing emphasis on integrating innovations and best practices in anesthesia to optimize perioperative outcomes (25). Despite the promising role of dexmedetomidine in orthognathic surgery, the absence of ERAS protocols specifically tailored to oral and maxillofacial surgery, particularly for orthognathic procedures, represents a significant gap in the field (4). Standardizing protocols for preoperative preparation, multimodal analgesia, and postoperative recovery is essential to improve clinical outcomes and minimize variability in patient care.

Additionally, effective patient communication and expectation management are fundamental to enhancing postoperative satisfaction, yet these aspects remain underexplored in the context of dexmedetomidine use (26). Future efforts should prioritize the development of a comprehensive, evidence-based ERAS protocol for orthognathic surgery, ideally established through consensus conferences and structured clinical implementation, to enhance both patient recovery and healthcare efficiency (4).

Effective postoperative pain management remains a significant challenge in orthognathic surgery, as patients often experience considerable discomfort due to extensive bone and soft tissue manipulation. This review highlights the promising role of dexmedetomidine in reducing postoperative pain and analgesic requirements, reinforcing its potential as a key component of multimodal pain management strategies. Given that orthognathic surgery is frequently performed in patients with obstructive sleep apnea syndrome, who may have heightened pain sensitivity and disrupted sleep patterns, optimizing perioperative analgesia is crucial (27).

Preoperative sleep quality has been identified as a modifiable factor that may enhance postoperative pain control, further emphasizing the need for a comprehensive perioperative care approach (28). Given that maxillomandibular advancement surgery significantly improves overall quality of life, effective pain management becomes essential to maximize patient satisfaction and facilitate recovery (29). These findings underscore the need for future research to integrate of dexmedetomidine into standardized multimodal pain management protocols, particularly to determine the most effective dosage and duration for its administration (24).

Although prolonged extubation time and potential cardiovascular complications have been associated with dexmedetomidine when used as an opioid substitute in opioid-free anesthesia (12), these concerns were not as prominent in the findings of this systematic review. As a sedative and hypotensive agent, dexmedetomidine’s dosage in the eligible studies may not have been sufficient to produce the same adverse effects observed at higher doses when used as an opioid substitute. Furthermore, investigating preoperative biomarkers to predict cardiovascular events could be valuable, as demonstrated in orthopedic surgery studies (30). Future research should explore this avenue to enhance patient safety and optimize perioperative risk stratification.

Moreover, the distinct effects of dexmedetomidine on heart rate could inadvertently reveal its administration to experienced anesthesiologists, even in blinded clinical trials, potentially introducing bias in study outcomes (21). Bradycardia is an expected occurrence in hypotensive anesthesia, and anesthesiologists should be well-prepared to implement multimodal protocols to ensure hemodynamic stability. The selection of these protocols should be guided by a comprehensive assessment of inherent risks and a thorough cost-benefit analysis (19).

This systematic review has some limitations. The small sample size and high heterogeneity among the included studies restricted the feasibility of conducting a meta-analysis and limited the strength of the conclusions. Additionally, the scarcity of high-quality randomized clinical trials on this topic reflects a broader trend in surgical research, where case reports and case series are more frequently published than robust clinical studies. The lack of standardized administration protocols and dosage regimens for dexmedetomidine further complicates result comparisons, hindering the identification of the most effective and safest perioperative approach in orthognathic surgery. Furthermore, the absence of detailed information on certain medication protocols may also introduce a significant source of bias, as potential pharmacological interactions could confound the measured outcomes. Variability in study designs, outcome measures, and patient selection criteria also contributes to inconsistencies in the available evidence.

Despite these limitations, this systematic review presents several strengths. It synthesized the current literature on dexmedetomidine use in orthognathic surgery, adhering to Evidence-Based Practice principles, even with the limited number of randomized clinical trials. The review identified clinically relevant perioperative events associated with dexmedetomidine, which provide a foundation for future research and further clinical trials. Additionally, dexmedetomidine demonstrated promising potential as a hypotensive agent in orthognathic surgery, provided that its risk-benefit profile is carefully evaluated by anesthesiologists on a case-by-case basis. The systematic search strategy was comprehensive, covering multiple databases and adhering to rigorous methodological standards to ensure high-quality evidence and minimize selection bias. These strengths enhance the reliability of the findings and underscore key areas for future investigation, particularly in optimizing multimodal analgesia, standardizing ERAS protocols, and refining cardiovascular risk assessment strategies.

## Conclusions

Based on available evidence, the use of dexmedetomidine in hypotensive anesthesia for patients undergoing orthognathic surgery demonstrates potential benefits in reducing postoperative symptoms such as pain, nausea, vomiting, and cough while maintaining hemodynamic stability. Dexmedetomidine appears to be a safe and effective option in this context. However, anesthesiologists should carefully evaluate its risk-benefit profile on a case-by-case basis. Further research is needed to strengthen evidence on ERAS protocols in orthognathic surgery and optimize perioperative management.

## Figures and Tables

**Table 1:** Databases search strategies.

Databases	Search strategies (December 2023) and Update (January 2025)
Main databases	
Cochrane Library https://www.cochranelibrary.com/	#1 "Hypnotics and Sedatives" OR "Sedative" OR "Hypnotic" OR "Dexmedetomidine" OR "MPV1440" OR "Precedex" OR "Dexdomitor" OR "Sileo" OR "Dexdor" OR "Dexmedetomidine Hydrochloride" OR "Igalmi" OR "Hydrochloride, Dexmedetomidine" OR "Preanesthetic Medication" OR "Medication, Preanesthetic" OR "Preanesthetic Medications"
	#2 "Orthognathic Surgery" OR "Orthognathic Surgeries" OR "Surgery, Orthognathic" OR "Orthognathic Surgical Procedures" OR "Orthognathic Surgical Procedure" OR "Surgical Procedures, Orthognathic" OR "Jaw Surgery" OR "Jaw Surgeries" OR "Surgeries, Jaw" OR "Surgery, Jaw" OR "Maxillo Mandibular Surgery" OR "Surgery, Maxillo-Mandibular" OR "Maxillofacial Orthognathic Surgery" OR "Maxillofacial Orthognathic Surgeries" OR "Orthognathic Surgery, Maxillofacial"
	#1 AND #2
Embase https://www.embase.com	(‘hypnotics and sedatives'/exp OR ‘hypnotics and sedatives' OR ‘sedative'/exp OR ‘sedative' OR ‘hypnotic' OR ‘dexmedetomidine'/exp OR ‘dexmedetomidine' OR ‘mpv1440'/exp OR ‘mpv1440' OR ‘precedex'/exp OR ‘precedex' OR ‘dexdomitor'/exp OR ‘dexdomitor' OR ‘sileo'/exp OR ‘sileo' OR ‘dexdor'/exp OR ‘dexdor' OR ‘dexmedetomidine hydrochloride'/exp OR ‘dexmedetomidine hydrochloride' OR ‘igalmi'/exp OR ‘igalmi' OR ‘hydrochloride, dexmedetomidine' OR ‘preanesthetic medication'/exp OR ‘preanesthetic medication' OR ‘medication, preanesthetic' OR ‘preanesthetic medications') AND (‘orthognathic surgery'/exp OR ‘orthognathic surgery' OR ‘orthognathic surgeries' OR ‘surgery, orthognathic' OR ‘orthognathic surgical procedures'/exp OR ‘orthognathic surgical procedures' OR ‘orthognathic surgical procedure' OR ‘surgical procedures, orthognathic' OR ‘jaw surgery'/exp OR ‘jaw surgery' OR ‘jaw surgeries' OR ‘surgeries, jaw' OR ‘surgery, jaw' OR ‘maxillo mandibular surgery' OR ‘surgery, maxillo-mandibular' OR ‘maxillofacial orthognathic surgery' OR ‘maxillofacial orthognathic surgeries' OR ‘orthognathic surgery, maxillofacial')
LILACS http://lilacs.bvsalud.org/	("Hypnotics and Sedatives" OR "Sedative" OR "Hypnotic" OR "Dexmedetomidine" OR "MPV1440" OR "Precedex" OR "Dexdomitor" OR "Sileo" OR "Dexdor" OR "Dexmedetomidine Hydrochloride" OR "Igalmi" OR "Hydrochloride, Dexmedetomidine" OR "Preanesthetic Medication" OR "Medication, Preanesthetic" OR "Preanesthetic Medications") AND ("Orthognathic Surgery" OR "Orthognathic Surgeries" OR "Surgery, Orthognathic" OR "Orthognathic Surgical Procedures" OR "Orthognathic Surgical Procedure" OR "Surgical Procedures, Orthognathic" OR "Jaw Surgery" OR "Jaw Surgeries" OR "Surgeries, Jaw" OR "Surgery, Jaw" OR "Maxillo Mandibular Surgery" OR "Surgery, Maxillo-Mandibular" OR "Maxillofacial Orthognathic Surgery" OR "Maxillofacial Orthognathic Surgeries" OR "Orthognathic Surgery, Maxillofacial") AND ( db:("LILACS"))
MEDLINE (via PubMed) http://www.ncbi.nlm.nih.gov/pubmed	#1 "Hypnotics and Sedatives"[Mesh] OR "Sedative"[tw] OR "Hypnotic"[tw] OR "Dexmedetomidine"[Mesh] OR "MPV1440"[tw] OR "Precedex"[tw] OR "Dexdomitor"[tw] OR "Sileo"[tw] OR "Dexdor"[tw] OR "Dexmedetomidine Hydrochloride"[tw] OR "Hydrochloride, Dexmedetomidine"[tw] OR "Igalmi"[tw] OR "Preanesthetic Medication"[Mesh] OR "Medication, Preanesthetic"[tw] OR "Preanesthetic Medications"[tw]
	#2 "Orthognathic Surgery"[Mesh] OR "Orthognathic Surgeries"[tw] OR "Surgery, Orthognathic"[tw] OR "Orthognathic Surgical Procedures"[Mesh] OR "Orthognathic Surgical Procedure"[tw] OR "Surgical Procedures, Orthognathic"[tw] OR "Jaw Surgery"[tw] OR "Jaw Surgeries"[tw] OR "Surgeries, Jaw"[tw] OR "Surgery, Jaw"[tw] OR "Maxillo Mandibular Surgery"[tw] OR "Surgery, Maxillo-Mandibular"[tw] OR "Maxillofacial Orthognathic Surgery"[tw] OR "Maxillofacial Orthognathic Surgeries"[tw] OR "Orthognathic Surgery, Maxillofacial"[tw]
	#1 AND #2
SciELO https://scielo.org/	#1 "Hypnotics and Sedatives" OR "Sedative" OR "Hypnotic" OR "Dexmedetomidine" OR "MPV1440" OR "Precedex" OR "Dexdomitor" OR "Sileo" OR "Dexdor" OR "Dexmedetomidine Hydrochloride" OR "Igalmi" OR "Hydrochloride, Dexmedetomidine" OR "Preanesthetic Medication" OR "Medication, Preanesthetic" OR "Preanesthetic Medications"
	#2 "Orthognathic Surgery" OR "Orthognathic Surgeries" OR "Surgery, Orthognathic" OR "Orthognathic Surgical Procedures" OR "Orthognathic Surgical Procedure" OR "Surgical Procedures, Orthognathic" OR "Jaw Surgery" OR "Jaw Surgeries" OR "Surgeries, Jaw" OR "Surgery, Jaw" OR "Maxillo Mandibular Surgery" OR "Surgery, Maxillo-Mandibular" OR "Maxillofacial Orthognathic Surgery" OR "Maxillofacial Orthognathic Surgeries" OR "Orthognathic Surgery, Maxillofacial"
	#1 AND #2
Scopus http://www.scopus.com/	( TITLE-ABS-KEY ( "Hypnotics and Sedatives" OR "Sedative" OR "Hypnotic" OR "Dexmedetomidine" OR "MPV1440" OR "Precedex" OR "Dexdomitor" OR "Sileo" OR "Dexdor" OR "Dexmedetomidine Hydrochloride" OR "Igalmi" OR "Hydrochloride, Dexmedetomidine" OR "Preanesthetic Medication" OR "Medication, Preanesthetic" OR "Preanesthetic Medications" ) AND TITLE-ABS-KEY ( "Orthognathic Surgery" OR "Orthognathic Surgeries" OR "Surgery, Orthognathic" OR "Orthognathic Surgical Procedures" OR "Orthognathic Surgical Procedure" OR "Surgical Procedures, Orthognathic" OR "Jaw Surgery" OR "Jaw Surgeries" OR "Surgeries, Jaw" OR "Surgery, Jaw" OR "Maxillo Mandibular Surgery" OR "Surgery, Maxillo-Mandibular" OR "Maxillofacial Orthognathic Surgery" OR "Maxillofacial Orthognathic Surgeries" OR "Orthognathic Surgery, Maxillofacial" ) )
Web of Science http://apps.webofknowledge.com/	#1 TS=("Hypnotics and Sedatives" OR "Sedative" OR "Hypnotic" OR "Dexmedetomidine" OR "MPV1440" OR "Precedex" OR "Dexmedetomidine Hydrochloride" OR "Hydrochloride, Dexmedetomidine" OR "Preanesthetic Medication" OR "Medication, Preanesthetic" OR "Preanesthetic Medications")
	#2 TS=("Orthognathic Surgery" OR "Orthognathic Surgeries" OR "Surgery, Orthognathic" OR "Orthognathic Surgical Procedures" OR "Orthognathic Surgical Procedure" OR "Surgical Procedures, Orthognathic" OR "Jaw Surgery" OR "Jaw Surgeries" OR "Surgeries, Jaw" OR "Surgery, Jaw" OR "Maxillo Mandibular Surgery" OR "Surgery, Maxillo-Mandibular" OR "Maxillofacial Orthognathic Surgery" OR "Maxillofacial Orthognathic Surgeries" OR "Orthognathic Surgery, Maxillofacial")
	#1 AND #2
Gray literature	
EASY https://easy.dans.knaw.nl/	"Dexmedetomedine" OR "Precedex" OR "Sedative" OR "Hypnotic"

**Table 2:** Main characteristics of eligible studies.

Author, year (Country)	Perioperative medication protocols	Anesthesia protocols			Perioperative events assessed and assessment methods
		Preparation	Maintenance	Recovery
Journal of publication (Impact factor)				
Study design				
Sample (♀, ♂)				
Age range				
Farah et al., 2008 (Brazil)	Preanesthetic (Midazolam 15mg po 60min before procedure); Volemic reposition (Glucose serum 5% 1mL/Kg/h of fasting + Ringer's lactate 4mL/Kg/h + Ringer's lactate 2.5mL/mL of lost blood); Analgesia (Ketoprofen 100mg IV stat before procedure + Tramadol 1mg/Kg IV stat after procedure); Anti-emetics (Dexamethasone 10mg IV stat before procedure + Ondansetron 4 mg IV stat after procedure); Antimicrobials (Metronidazole 500mg IV stat before procedure + Cefazoline 2g IV stat before procedure + Cefazoline 1g IV 3/3h after procedure); Local anesthesia (lidocaine 2% with epinephrine 1:100,000 UI 10mL at max); Low mean arterial pressure (Esmolol 0.5mg/kg IV for 60min + Esmolol 100-to-200mcg/kg/min); Other specific perioperative medication protocols NR.	Intervention group: Dexmedetomidine 1mcg/Kg 2mg in 100mL of physiological serum 0,9% 5-to-20min before induction + Sufentanyl 0.5mcg/Kg + Propofol 2mg/Kg + Pancuronium 0.1mcg/Kg.	Intervention group: Propofol 3mcg/mL + Isoflurane 0.5-to-1.0 minimum alveolar concentration + Dexmedetomidine 0.3-to-0.5mcg/Kg/h + Pancuronium in fractioned doses.	Intervention group: Propofol suspended 20min before the end of procedure + Atropine 0.02mg/Kg 2mg at max + Prostigmine 0.04mg/Kg 4mg at max.	Extubation time per minute; Nausea and vomiting per incidence; Atropine and esmolol use per incidence, blood pressure, temperature and heart rate; PACU time per minute; Awakening time per minute; Pain per incidence using VAS scores.
J Oral Maxillofac Surg (2.3)					
RCT, prospective					
		Control group: Clonidine 1-to-2mcg/Kg in 100mL of physiological serum 0,9% 5-to-20min before induction + Sufentanyl 0.5mcg/Kg + Propofol 2mg/Kg + Pancuronium 0.1mcg/Kg.	Control group: Propofol 3mcg/mL + Remifentanil 0.1-to-0.3mcg/Kg/min + Pancuronium in fractioned doses.	Control group: Propofol suspended 20min before the end of procedure + Remifentanil suspended at the end of procedure + Atropine 0.02mg/Kg 2mg at max + Prostigmine 0.04mg/Kg 4mg at max.	
20 (14♀, 6♂)					
17-44 yo					
Ham et al., 2014 (South Korea)	Preanesthetic (Glycopyrrolate 0.1mg + Rocuronium 0.6mg/kg); Analgesia (Ketorolac 1mg/kg + Fentanyl 1mcg/kg); Anti-emetic (Ondansetron 4mg); Emergence agitation (Midazolam 0.05mcg/kg); Low mean arterial pressure (Nicardipine IV or Esmolol IV); Other specific perioperative medication protocols NR.	Intervention group: Propofol 2mg/kg + Desflurane + Remifentanil 0.2-to-0.5mcg/kg.	Intervention group: Desflurane 1.0 minimum alveolar concentration + Remifentanil 0.05-to-2mcg/kg/min + Phenylephrine 20-to-50mcg + Dexmedetomidine 4mcg/mL IV for 10min stat after suturing started.	Intervention group: Glycopyrrolate + Neostigmine + Desflurane suspended + Oxygen flow increased to 6L/min + Remifentanil 0.02mcg/kg/min IV suspended after eye opening.	Cough per four-point scale and severe cough defined as ≥ 2 points; Respiratory rate per minute and end-tidal CO_2_ concentration per kPA; Rescue antiemetics per incidence; Phenylephrine used per incidence, blood pressure and heart rate; Time to discharge from OR per minute; Emergence agitation per incidence using RASS ≥ +2 and severe emergence agitation using RASS ≥ +3; Eye opening time per minute and residual sedation per incidence using RASS ≤ -2; Pain using NRS scores.
Acta Anaesthesiol Scand (1.9)					
RCT, double-blinded, placebo-controlled, prospective					
		Control group: Propofol 2mg/kg + Desflurane + Remifentanil 0.2-to-0.5mcg/kg.	Control group: Desflurane 1.0 minimum alveolar concentration + Remifentanil 0.05-to-2mcg/kg/min + Phenylephrine 20-to-50mcg + Normal saline IV for 10min stat after suturing started.	Control group: Glycopyrrolate + Neostigmine + Desflurane suspended + Oxygen flow increased to 6L/min + Remifentanil 0.02mcg/kg/min IV suspended after eye opening.	
70 (41♀, 29♂)					
20-45 yo					
Rummasak & Apipan, 2014 (Thailand)	Analgesia (Meperidine 25mg after procedure); Other specific perioperative medication protocols NR.	Intervention group: Dexmedetomidine 1mcg/kg for 15min stat before procedure + Midazolam 0.05mg/kg IV + Fentanyl 1mcg/kg IV + Propofol 2mg/kg IV.	Intervention group: Dexmedetomidine 0.2-to-0.7 mcg/kg per hour + Nitrous oxide in oxygen + Cis-atracurium 1-to-2 twitches of the train-of-four + Fentanyl 1mcg/kg + Sevoflurane 1-to-3% directed by bispectral index.	Intervention group: Dexmedetomidine suspended stat after procedure + Sevoflurane and Nitrous oxide suspended + Oxygen flow increased to 6L/min + Neostigmine 0.05mg/kg + Atropine 0.02mg/kg.	Extubation time per minute; Blood pressure, pulse, blood loss per mL, and hemoglobin per g/dL; Eye opening and time to follow commands per minute; Pain using NRS scores (at 30 and 60-min postop), and meperidine used per mg.
J Oral Maxillofac Surg (2.3)					
RCT, single-blinded, prospective					
		Control group: Nitroglycerin 10-to-20mcg/kg titration + Midazolam 0.05mg/kg IV + Fentanyl 1mcg/kg IV + Propofol 2mg/kg IV.	Control group: Nitroglycerin increased titration every 3-to-5min, 400mcg/minute at max + Nitrous oxide in oxygen + Cis-atracurium 1-to-2 twitches of the train-of-four + Fentanyl 1mcg/kg + Sevoflurane 1-to-3% directed by bispectral index.	Control group: Nitroglycerin suspended stat after procedure + Sevoflurane and Nitrous oxide suspended + Oxygen flow increased to 6L/min + Neostigmine 0.05mg/kg + Atropine 0.02mg/kg.	
40 (26♀, 14♂)					
18-45 yo					
Shin et al., 2014 (South Korea)	No patients received premedication; Nasotracheal intubation (Vecuronium 0.15mg/kg after maintenance of end-tidal sevoflurane concentration of 5% for at least 5min); Other specific perioperative medication protocols NR.	Intervention group: Propofol 2mg/kg IV + Sevoflurane inhalation 5%.	Intervention group: Sevoflurane inhalation 1-to-2% + Air 50% in oxygen 8mL/kg + Dexmedetomidine 1mcg/kg loaded for 10min stat after surgery started, followed by infusion 0.2-to-1mcg/kg/h.	Specific anesthesia recovery protocols NR.	Intraoperative fluid, transfusion, urine output and estimated blood loss in mL; Blood pressure, heart rate and QT intervals; Consciousness using bispectral index score; End-tidal sevoflurane concentration.
Acta Anaesthesiol Scand (1.9)					
RCT, single-blinded, prospective					
62 (28♀, 34♂)					
Age range NR.					
		Control group 1: Propofol 2mg/kg IV + Sevoflurane inhalation 5%.	Control group 1: Sevoflurane inhalation 1-to-2% + Air 50% in oxygen 8mL/kg + Nicardipine 1-to-7mcg/kg/min IV stat after surgery started.		
		Control group 2: Propofol 2mg/kg IV + Sevoflurane inhalation 5%.	Control group 2: Sevoflurane inhalation 1-to-2% + Air 50% in oxygen 8mL/kg + Remifentanil 0.05-to-2mcg/kg/min IV stat after surgery started.		
			No other adjuvant drugs were administered during controlled hypotension for all groups.		
Goswami et al., 2022 (India)	Preanesthetic (Midazolam 1mg); Analgesia (Paracetamol 15mg/kg IV + Ketorolac 0.5mg/kg IV); Bradycardia (Glycopyrrolate 0.2mg IV); Low mean arterial pressure (Ephedrine 6mg IV); Other specific perioperative medication protocols NR.	Intervention group: Dexmedetomidine 1mcg/kg for 10min + Propofol 2mg/kg + Fentanyl 2mcg/kg + Vecuronium 0.1mg/kg.	Intervention group: Dexmedetomidine 0.2-to-0.5mcg/kg/h + Desflurane + Vecuronium + Fentanyl 1mcg/kg.	Intervention group: Dexmedetomidine suspended 20min stat before procedure's end.	Bradycardia, hypotensive episode, difficulty to achieve hypotension, blood transfusion per incidence, and blood loss per mL; Rescue analgesics per mcg.
J Oral Maxillofac Surg (2.3)					
RCT, double-blinded, prospective					
		Control group: Clonidine 0.3mcg/kg for 10min + Propofol 2mg/kg + Fentanyl 2mcg/kg + Vecuronium 0.1mg/kg.	Control group: Clonidine 0.3-to-2.0mcg/kg/h + Desflurane + Vecuronium + Fentanyl 1mcg/kg.	Control group: Clonidine suspended 20min stat before procedure's end.	
30 (16♀, 14♂)					
18-25 yo					
Labafchi et al., 2023 (Iran)	Preanesthetic (Midazolam 2mg IV + Fentanyl 0.01mcg/kg/min IV + Atracurium 0.15mg/kg IV); Volemic reposition (glucose-saline serum 1:2 IV within 6h postop); Analgesia (Acetaminophen 1g IV 6/6h postop + Diclofenac 100mg 12/12h suppository postop); Antibiotic (Cephazolin 1g IV 6/6 postop); Anti-emetic (Metoclopramide 10mg IV); Bradycardia (Fentanyl 50mg); Edema (Dexamethasone 8mg IV 1h preop, 4/4h intraop, 8/8h postop); Other specific perioperative medication protocols NR.	Intervention group: Dexmedetomidine 1mcg/kg IV for 10min 20min before procedure + Propofol 2mg/kg.	Intervention group: Dexmedetomidine 0.2mcg/kg/h + Propofol 100-to-200mg/kg/min.	Intervention group: Neostigmine 0.004mg/kg IV + Atropine 0.02mg/kg IV.	Nausea and vomiting per incidence; Pain using VAS at six evaluation time points (1, 3, 6, 12, 18, and 24-hours postop) and rescue analgesics per incidence.
J Oral Maxillofac Surg (2.3)					
		Control group: Clonidine 0.3mcg/kg for 10min + Propofol 2mg/kg + Fentanyl 2mcg/kg + Vecuronium 0.1mg/kg.	Control group: Clonidine 0.3-to-2.0mcg/kg/h + Desflurane + Vecuronium + Fentanyl 1mcg/kg.	Control group: Clonidine suspended 20min stat before procedure's end.	
RCT, triple-blinded, placebo-controlled, prospective					
60 (38♀, 22♂)					
18-25 yo					

NR - not reported in the study; RCT - Randomized Clinical Trial; po - Per Oralis, oral administration; IV - Intravenous administration; Stat - Statim, immediately; At max - At maximum; Preop - Preoperatively; Postop - Postoperatively; PACU - Post-Anesthesia Care Unit; VAS - Visual Analog Scale; RASS- Richmond Agitation-Sedation Scale; NRS - Numerical Rating Scale; YO: year old.

**Table 3:** Main results of eligible studies.

Author, year -	Groups (n)	Results of outcomes	Statistical tests	p value
Measurement of outcomes				
Airway and Respiratory Events				
Farah et al., 2008 - Extubation time per min	Dexmedetomidine (n=10)	34.1 min	Descriptive analysis	N/A
	Clonidine (n=10)	24.5 min		
Ham et al., 2014 - Cough in OR per incidence	Dexmedetomidine (n=34)	16 cases	Chi-square or Fisher's exact test	0.24
	Normal saline (n=36)	22 cases		
Ham et al., 2014 - Severe cough in OR per incidence	Dexmedetomidine (n=34)	9 cases	Chi-square or Fisher's exact test	0.04*
	Normal saline (n=36)	18 cases		
Ham et al., 2014 - Cough at PACU per incidence	Dexmedetomidine (n=34)	2 cases	Chi-square or Fisher's exact test	0.004*
	Normal saline (n=36)	12 cases		
Ham et al., 2014 - Respiratory rate per min	Dexmedetomidine (n=34)	At eye opening^a^: 16±4 min	One-Way ANOVA	0.33^a^; 0.31^b^; 0.59^c^
		OR discharge^b^: 16±4 min		
		PACU discharge^c^: 16±6 min		
	Normal saline (n=36)	At eye opening^a^: 14±5 min		
		OR discharge^b^: 15±4 min		
		PACU discharge^c^: 17±4 min		
Ham et al., 2014 - End-tidal CO_2_ concentration in kPa	Dexmedetomidine (n=34)	At eye opening^a^: 5.3±0.5 kPa	One-Way ANOVA	0.04^a^*; 0.01^b^*; 0.97^c^
		OR discharge^b^: 5.3±0.5 kPa		
		PACU discharge^c^: 4.5±0.8 kPa		
	Normal saline (n=36)	At eye opening^a^: 5.6±0.7 kPa		
		OR discharge^b^: 5.7±0.7 kPa		
		PACU discharge^c^: 4.5±0.4 kPa		
Rummasak & Apipan, 2014 - Extubation time per min	Dexmedetomidine (n=20)	8.30±3.23 min	Student's t-test	0.19
	Nitroglycerin (n=20)	7.00±2.92 min		
Emetic Events				
Farah et al., 2008 - Nausea / vomiting per incidence	Dexmedetomidine (n=10)	0 nausea / 4 vomiting cases	Descriptive analysis	N/A
	Clonidine (n=10)	4 nausea / 0 vomiting cases		
Ham et al., 2014 - Rescue antiemetics per incidence	Dexmedetomidine (n=34)	4 cases	Chi-square or Fisher's exact test	0.22
	Normal saline (n=36)	2 cases		
Labafchi et al., 2023 - Nausea / vomiting per incidence	Dexmedetomidine (n=30)	1 nausea / 0 vomiting cases	One-Way ANOVA	< 0.001*
	Clonidine (n=30)	14 nausea / 0 vomiting cases		
Hemodynamic Events				
Farah et al., 2008 - Atropine / esmolol use per incidence	Dexmedetomidine (n=10)	2 atropine / 2 esmolol cases	Descriptive analysis	N/A
	Clonidine (n=10)	0 atropine / 10 esmolol cases		
Farah et al., 2008 - Blood pressure, temperature and heart rate	Dexmedetomidine (n=10)	No significant differences reported	One-Way ANOVA	> 0.05
	Clonidine (n=10)	No significant differences reported		
Ham et al., 2014 - Phenylephrine use per incidence	Dexmedetomidine (n=34)	26 cases	Chi-square or Fisher's exact test	< 0.0001*
	Normal saline (n=36)	4 cases		
Ham et al., 2014 - Blood pressure and heart rate	Dexmedetomidine (n=34)	Attenuated elevation of heart rate	One-Way ANOVA	< 0.05*
		Lower mean arterial pressure		
	Normal saline (n=36)	Elevated heart rate		
		Higher mean arterial pressure		
Rummasak & Apipan, 2014 - Systolic blood pressure in mmHg	Dexmedetomidine (n=20)	115.25±9.89 mmHg	Student's t-test	0.98
	Nitroglycerin (n=20)	115.15±12.07 mmHg		
Rummasak & Apipan, 2014 - Diastolic blood pressure in mmHg	Dexmedetomidine (n=20)	69.10±7.36 mmHg	Student's t-test	0.11
	Nitroglycerin (n=20)	73.55±9.48 mmHg		
Rummasak & Apipan, 2014 - Pulse in bpm	Dexmedetomidine (n=20)	77.65±10.42 bpm	Student's t-test	0.69
	Nitroglycerin (n=20)	76.20±12.63 bpm		
Rummasak & Apipan, 2014 - Blood loss in mL	Dexmedetomidine (n=20)	695.00±314.52 mL	Student's t-test	0.43
	Nitroglycerin (n=20)	785.00±391.05 mL		
Rummasak & Apipan, 2014 - Hemoglobin in g/dL	Dexmedetomidine (n=20)	Preop^a^: 13.29±1.29 g/dL	Student's t-test	0.53^a^; 0.33^b^
		1-day postop^b^: 10.58±1.22 g/dL		
	Nitroglycerin (n=20)	Preop^a^: 13.57±1.53 g/dL		
		1-day postop^b^: 11.59±4.41g/dL		
Shin et al., 2014 - Intraoperative fluid in mL	Dexmedetomidine (n=20)	1717.5±418.4 mL	One-Way ANOVA with Bonferroni's correction	0.327
	Nicardipine (n=21)	1995.2±627.7 mL		
	Remifentanil (n=21)	1931.0±743.4 mL		
Shin et al., 2014 - Intraoperative transfusion in mL	Dexmedetomidine (n=20)	156.5±201.3 mL	One-Way ANOVA with Bonferroni's correction	0.654
	Nicardipine (n=21)	211.7±197.7 mL		
	Remifentanil (n=21)	161.2±237.8 mL		
Shin et al., 2014 - Intraoperative urine output in mL	Dexmedetomidine (n=20)	348.8±197.9 mL	One-Way ANOVA with Bonferroni's correction	0.207
	Nicardipine (n=21)	287.6±108.0 mL		
	Remifentanil (n=21)	388.6±223.9 mL		
Shin et al., 2014 - Estimated blood loss in mL	Dexmedetomidine (n=20)	620.0±122.9 mL	One-Way ANOVA with Bonferroni's correction	0.14
	Nicardipine (n=21)	790.5±281.8 mL		
	Remifentanil (n=21)	695.2±351.7 mL		
Shin et al., 2014 - Heart rate in bpm	Dexmedetomidine (n=20)	T1 after induction: 79.1±13.0	Wilcoxon signed-rank test within groups (*); Kruskal-Wallis' test with Bonferroni's correction between groups (†)	< 0.05*; < 0.05†
		T2 during maintenance: 69.2±5.6*†		
	Nicardipine (n=21)	T1 after induction: 80.7±10.7		
		T2 during maintenance: 114.0±9.5*		
	Remifentanil (n=21)	T1 after induction: 80.1±13.0		
		T2 during maintenance: 67.5±7.6*†		
Shin et al., 2014 - Blood pressure in mmHg	Dexmedetomidine (n=20)	T1 after induction: 77.5±8.3	Paired t-test	< 0.05*
		T2 during maintenance: 61.3±3.0*		
	Nicardipine (n=21)	T1 after induction: 79.6±5.8		
		T2 during maintenance: 60.7±3.1*		
	Remifentanil (n=21)	T1 after induction: 76.6±5.5		
		T2 during maintenance: 61.0±3.0*		
Goswami et al., 2022 - Bradycardia per incidence	Dexmedetomidine (n=15)	0 cases	Descriptive analysis	N/A
	Clonidine (n=15)	1 case		
Goswami et al., 2022 - Hypotensive episode per incidence	Dexmedetomidine (n=15)	1 case	Descriptive analysis	N/A
	Clonidine (n=15)	3 cases		
Goswami et al., 2022 - Initial difficulty to achieve targeted hypotension per incidence	Dexmedetomidine (n=15)	0 cases	Descriptive analysis	N/A
	Clonidine (n=15)	1 case		
Goswami et al., 2022 - Blood transfusion per incidence	Dexmedetomidine (n=15)	1 case	Descriptive analysis	N/A
	Clonidine (n=15)	0 cases		
Goswami et al., 2022 - Blood loss in mL	Dexmedetomidine (n=15)	316.66±147.19 mL	Unpaired t-test	0.716
	Clonidine (n=15)	263.33±112.54 mL		
Length of Hospital Stay				
Farah et al., 2008 - PACU time per min	Dexmedetomidine (n=10)	55.8 min	Descriptive analysis	N/A
	Clonidine (n=10)	49.4 min		
Ham et al., 2014 - Time to discharge from OR per min	Dexmedetomidine (n=34)	14±5 min	Student's t-test	0.001*
	Normal saline (n=36)	10±3 min		
Neurological Events				
Farah et al., 2008 - Awakening time per min	Dexmedetomidine (n=10)	24.8 min	Descriptive analysis	N/A
	Clonidine (n=10)	18.5 min		
Ham et al., 2014 - Emergence agitation per incidence	Dexmedetomidine (n=34)	13 cases	Chi-square or Fisher's exact test	0.45
	Normal saline (n=36)	17 cases		
Ham et al., 2014 - Severe emergence agitation per incidence	Dexmedetomidine (n=34)	6 cases	Chi-square or Fisher's exact test	0.31
	Normal saline (n=36)	11 cases		
Ham et al., 2014 - Eye opening time per min	Dexmedetomidine (n=34)	11±4 min	Student's t-test	< 0.0001*
	Normal saline (n=36)	7±2 min		
Ham et al., 2014 - Residual sedation per incidence	Dexmedetomidine (n=34)	4 cases	Chi-square or Fisher's exact test	0.19
	Normal saline (n=36)	1 case		
Rummasak & Apipan, 2014 - Eye opening time per min	Dexmedetomidine (n=20)	6.10±3.06 min	Student's t-test	0.046*
	Nitroglycerin (n=20)	4.40±2.04 min		
Rummasak & Apipan, 2014 - Time to follow commands per min	Dexmedetomidine (n=20)	6.20±2.93 min	Student's t-test	0.041*
	Nitroglycerin (n=20)	4.55±1.91 min		
Shin et al., 2014 - Bispectral index score (0-to-100)	Dexmedetomidine (n=20)	T1 after induction: 47.5±7.6	Paired t-test	> 0.05
		T2 during maintenance: 40.0±8.2		
	Nicardipine (n=21)	T1 after induction: 43.3±5.8		
		T2 during maintenance: 40.8±10.4		
	Remifentanil (n=21)	T1 after induction: 44.1±5.2		
		T2 during maintenance: 41.9±5.1		
Shin et al., 2014 - End-tidal sevoflurane concentration in %	Dexmedetomidine (n=20)	T1 after induction: 1.8±0.3	Paired t-test	< 0.05*
		T2 during maintenance: 2.4±0.4*		
	Nicardipine (n=21)	T1 after induction: 1.8±0.2		
		T2 during maintenance: 2.3±0.2*		
	Remifentanil (n=21)	T1 after induction: 1.7±0.2%		
		T2 during maintenance: 2.2±0.3*		
Pain Burden				
Farah et al., 2008 - VAS 3-score / 5-score per incidence	Dexmedetomidine (n=10)	1 3-score / 1 5-score cases	Descriptive analysis	N/A
	Clonidine (n=10)	2 3-score / 2 5-score cases		
Ham et al., 2014 - NRS score (0-to-10)	Dexmedetomidine (n=34)	Median (Interquartile range): 0 (0, 2)	Mann-Whitney's t-test with Bonferroni's correction	0.046*
	Normal saline (n=36)	Median (Interquartile range): 2 (0, 4.5)		
Ham et al., 2014 - Rescue fentanyl per incidence	Dexmedetomidine (n=34)	2 cases	Chi-square or Fisher's exact test	0.10
	Normal saline (n=36)	1 case		
Rummasak & Apipan, 2014 - Rescue fentanyl in mcg	Dexmedetomidine (n=20)	168.75±56.29 mcg	Student's t-test	0.037*
	Nitroglycerin (n=20)	222.50±96.12 mcg		
Rummasak & Apipan, 2014 - NRS score (0-to-10)	Dexmedetomidine (n=20)	30-min postop: 4.80±2.59	Student's t-test	0.36; 0.36
		60-min postop: 4.40±1.66		
	Nitroglycerin (n=20)	30-min postop: 4.05±2.54		
		60-min postop: 3.90±1.77		
Rummasak & Apipan, 2014 -Meperidine use in mg	Dexmedetomidine (n=20)	18.75±13.75 mg	Student's t-test	0.15
	Nitroglycerin (n=20)	12.50±12.83 mg		
Goswami et al., 2022 - Rescue analgesics in mcg	Dexmedetomidine (n=15)	222±80.12 mcg	Unpaired t-test	0.25
	Clonidine (n=15)	254.66±73.95 mcg		
Labafchi et al., 2023 - VAS score (0-to-10)	Dexmedetomidine (n=30)	1h postop: 3.2±0.8	One-Way ANOVA	< 0.001*
		3h postop: 4.0±1.0		
		6h postop: 4.8±0.9		
		12h postop: 5.3±0.9		
		18h postop: 4.0±0.9		
		24h postop: 2.8±0.9		
	Clonidine (n=30)	1h postop: 4.2±0.9		
		3h postop: 4.8±0.9		
		6h postop: 5.7±0.9		
		12h postop: 6.6±1.4		
		18h postop: 5.1±1.1		
		24h postop: 3.8±0.8		
Labafchi et al., 2023 - Rescue analgesics per incidence	Dexmedetomidine (n=30)	2 cases	One-Way ANOVA	0.01*
	Clonidine (n=30)	10 cases		

N/A - Not applicable; OR - Operation Room; PACU - Post-Anesthesia Care Unit; VAS - Visual Analog Scale; NRS - Numeric Rating Scale; T1 - when vital signs were stable after anesthesia induction phase, outcomes were measured for 10min; T2 - when targeted hypotension was stable during anesthesia maintenance phase, outcomes were measured for 30min.

**Table 4:** Summary of findings table.

Outcomes	Main results	N. of patients (studies)	Certainty of evidence (GRADE)	Comments
Dexmedetomidine versus Clonidine on perioperative events of orthognathic surgery				
Extubation time per min	Similar time.	20 (1 RCT)	Very Low^a,b^	The evidence is very uncertain about the effect of dexmedetomidine compared with clonidine on preoperative events, with results indicating dexmedetomidine may decrease or have no effect on outcomes.
Nausea and vomiting per incidence	The dexmedetomidine group had significant less nausea cases than the clonidine group. In one study, dexmedetomidine group had more vomiting cases than clonidine group.	80 (2 RCT)		
Blood pressure, temperature and heart rate	No significant differences reported.	20 (1 RCT)		
PACU time per min	Similar time.	20 (1 RCT)		
Awakening time per min	Similar time.	20 (1 RCT)		
Pain (VAS score)	The dexmedetomidine group had significant less pain than the clonidine group.	80 (2 RCT)		
Pain (Rescue analgesics)	Both studies showed less use of analgesics in the dexmedetomidine group.	90 (2 RCT)	Low^b^	
Dexmedetomidine versus Saline solution on perioperative events of orthognathic surgery				
Cough in OR per incidence	The dexmedetomidine group had significant less severe cough than the saline group.	70 (1 RCT)	Low^b^	The evidence is uncertain about the effect of dexmedetomidine compared with saline solution on preoperative events, with results indicating dexmedetomidine may decrease or have no effect on outcomes.
Respiratory rate	No significant differences reported.			
Blood pressure and heart rate	The dexmedetomidine group attenuated elevation of heart rate and had lower mean arterial pressure.			
Time to discharge from OR per min	The dexmedetomidine group needed significant more time to discharge.			
Emergence agitation per incidence	No significant differences reported.			
Eye opening time per min	The dexmedetomidine group needed significant more time.			
Residual sedation per incidence	No significant differences reported.			
Pain (NRS score)	The dexmedetomidine group had significant less pain than the saline group.			
Dexmedetomidine versus Nitroglycerin on perioperative events of orthognathic surgery				
Extubation time per min	No significant differences reported.	40 (1 RCT)	Low^b^	The evidence is uncertain about the effect of dexmedetomidine compared with nitroglycerin on preoperative events, with results indicating dexmedetomidine may decrease or have no effect on outcomes.
Blood pressure, and heart rate	No significant differences reported.			
Eye opening time per min	The dexmedetomidine group needed significant more time.			
Time to follow commands per min	The dexmedetomidine group needed significant more time.			
Pain (NRS score)	No significant differences reported.			
Dexmedetomidine versus Nicardipine on perioperative events of orthognathic surgery				
Blood pressure	No significant differences reported.	41 (1 RCT)	Low^b^	The evidence is uncertain about the effect of dexmedetomidine compared with nicardipine on preoperative events, with results indicating dexmedetomidine may have no effect on outcomes.
Heart rate	No significant differences reported.			
Estimated blood loss	No significant differences reported.			
Bispectral index score	No significant differences reported.			
End-tidal sevoflurane concentration in %	No significant differences reported.			
Dexmedetomidine versus Remifentanil on perioperative events of orthognathic surgery				
Blood pressure	No significant differences reported.	41 (1 RCT)	Low^b^	The evidence is uncertain about the effect of dexmedetomidine compared with remifentanil on preoperative events, with results indicating dexmedetomidine may have no effect on outcomes.
Heart rate	No significant differences reported.			
Estimated blood loss	No significant differences reported.			
Bispectral index score	No significant differences reported.			
End-tidal sevoflurane concentration in %	No significant differences reported.			

Certainty of evidence was downgraded by one level due to some concerns in randomization and blindness. Certainty of evidence was downgraded by two levels because the total sample size was less than 100.
